# 3-*O*-Acetyloleanolic acid inhibits VEGF-A-induced lymphangiogenesis and lymph node metastasis in an oral cancer sentinel lymph node animal model

**DOI:** 10.1186/s12885-018-4630-0

**Published:** 2018-07-05

**Authors:** Jeon Hwang-Bo, Mun Gyeong Bae, Jong-Hwa Park, In Sik Chung

**Affiliations:** Department of Genetic Engineering and Graduate School of Biotechnology, Kyung Hee University, Yongin, 446-701 South Korea

**Keywords:** 3-*O*-acetyloleanolic acid, Lymphangiogenesis, Lymph node metastasis, Oral cancer sentinel lymph node animal model, VEGF-A

## Abstract

**Background:**

Sentinel lymph node metastasis is a common and early event in the metastatic process of head and neck squamous cell carcinoma (HNSCC) and is the most powerful prognostic factor for survival of HNSCC patients. 3-*O*-acetyloleanolic acid (3AOA), a pentacyclic triterpenoid compound isolated from seeds of *Vigna sinensis* K., has been reported to have potent anti-angiogenesis and anti-tumor activities. However, its effects on tumor-related lymphangiogenesis and lymph node metastasis are not yet understood.

**Methods:**

The in vitro inhibitory effects of 3AOA on VEGF-A-induced lymphangiogenesis were investigated via in vitro experiments using mouse oral squamous cell carcinoma (SCCVII) cells and human lymphatic microvascular endothelial cells (HLMECs). The in vivo inhibitory effects of 3AOA on VEGF-A-induced lymphangiogenesis and sentinel lymph node metastasis were investigated in an oral cancer sentinel lymph node (OCSLN) animal model.

**Results:**

3AOA inhibited tumor-induced lymphangiogenesis and sentinel lymph node metastasis in an OCSLN animal model, and reduced expression of VEGF-A, a lymphangiogenic factor in hypoxia mimetic agent CoCl_2_-treated SCCVII cells. 3AOA inhibited proliferation, tube formation, and migration of VEGF-A-treated HLMECs. The lymphatic vessel formation that was stimulated in vivo in a by VEGF-A Matrigel plug was reduced by 3AOA. 3AOA suppressed phosphorylation of vascular endothelial growth factor (VEGFR) -1 and − 2 receptors that was stimulated by VEGF-A. In addition, 3AOA suppressed phosphorylation of the lymphangiogenesis-related downstream signaling factors PI3K, FAK, AKT, and ERK1/2. 3AOA inhibited tumor growth, tumor-induced lymphangiogenesis, and sentinel lymph node metastasis in a VEGF-A-induced OCSLN animal model that was established using VEGF-A overexpressing SCCVII cells.

**Conclusion:**

3AOA inhibits VEGF-A-induced lymphangiogenesis and sentinel lymph node metastasis both in vitro and in vivo*.* The anti-lymphangiogenic effects of 3AOA are probably mediated via suppression of VEGF-A/VEGFR-1 and VEGFR-2 signaling in HLMECs, and can be a useful anti-tumor agent to restrict the metastatic spread of oral cancer.

**Electronic supplementary material:**

The online version of this article (10.1186/s12885-018-4630-0) contains supplementary material, which is available to authorized users.

## Background

Oral cancer, a kind of head and neck cancer, is any malignant tissue growth in the oral cavity. There are different types of oral cancers, more than 90% of which are squamous cell carcinoma [1]. Oral squamous cell carcinoma (OSCC) classification is based on disease stage. Standard care for OSCC includes a single treatment or a combination of surgery, irradiation, and chemotherapy. Unfortunately, the survival rate of OSCC patients has not improved significantly with time. New treatment methods for managing OSCC are required.

The main factor that affects the prognosis of patients with OSCC is regional lymph node metastasis, which usually occurs via the sentinel lymph node (SLN), the first lymph node draining from the primary tumor. Several studies have shown that metastasis from malignant tumors to lymph nodes occurs consistently, sequentially, and predictably. Therefore, accurate identification and histological examination of the sentinel lymph nodes plays an important role in diagnosis and treatment of malignant tumors [2]. Also, according to recent reports, the lymphatic system is more important than the vascular system in metastasis of head and neck squamous cell carcinoma (HNSCC) [3].

Lymphangiogenesis, a process of new lymphatic vessel formation from pre-existing lymphatic vessels, plays an important physiological and pathological role in embryonic development, wound healing, organ transplantation, tumor metastasis, and regeneration of tissues and organs [4]. Spreading of tumor cells from a primary tumor to lymph nodes via the lymphatic system is an early common event in metastasis, and lymphangiogenesis plays a critical role in promoting tumor spread to regional lymph nodes. Recent studies showed that tumor cells from several different malignancies can induce lymphangiogenesis in SLNs before metastasis, and that higher intratumoral lymphatic vessel and sentinel lymph node lymphatic vessel density values were significantly associated with the presence of lymph node metastases in patients. Changes in LNs begin before metastasis in a process termed tumor-reactive lymphadenopathy. Regional lymph nodes proximate to primary tumors are generally enlarged due to reactive lymphadenopathy, tumor metastasis, or both, suggesting that lymph nodes alteration results from interactions between the lymphatic system and tumors [5, 6].

Tumor-induced lymphangiogenensis is mediated by lymphangiogenic factors, such as vascular endothelial growth factors (VEGFs), fibroblast growth factor (FGF), angiopoietin-1 and angiopoietin-2, and platelet-derived growth factors (PDGFs) [7–9]. VEGF-C and VEGF-D are the main known lymphangiogenic factors that induce lymphangiogenesis through activation of vascular endothelial growth factor receptor (VEGFR) -3, the receptor for VEGF-C and VEGF-D that is expressed in LEC cells. Therefore, most experimentation in tumor-induced lymphangiogenesis related studies has focused on the roles of VEGF-C and VEGF-D in cancer progression [10]. However, it has recently been reported that VEGF-A, as well as VEGF-C and VEGF-D, acts as a lymphangiogenic factor in tumor-associated lymphangiogenesis and lymph node metastasis [11].

VEGF-A has been identified as the predominant angiogenic factor acting via VEGFR-1 and VEGFR-2. However, several recent studies have shown that VEGF-A promotes the proliferation and migration of human lymphatic endothelial cell in vitro [12–16], and it has been reported that targeted overexpression of VEGF-A acted to induce tumor lymphangiogenesis in cutaneous squamous cell carcinoma and promoted tumor spread to sentinel lymph nodes [5]. Also, our recent work has shown that VEGF-A is a lymphangiogenic factor expressed in SCCVII cells and oral squamous cell carcinomas under hypoxic conditions, and that VEGF-A promotes development of lymphatic vessels in vivo in a Matrigel plug [17].

3-*O*-acetyloleanolic acid (3AOA) is an oleanolic acid derivative and a pentacyclic triterpenoid compound isolated from the seeds of *Vigna sinensis K*.. Pentacyclic triterpenoids have exhibited a potent anti-tumor promotion activity during in vivo carcinogenesis testing, and exert cytotoxic activities against several cancer cell lines [18–20]. Oleanolic acid acts at different stages of tumorigenesis to suppress tumor initiation and promotion, as induces tumor cell differentiation and apoptosis. 3AOA induces apoptosis in human colon cancer (HCT-116) cells via the death receptor DR5-mediated caspase-8 activation cascade [21]. In our previous study, 3AOA isolated from cowpea seeds exhibited anti-angiogenic effects and induced apoptosis in human umbilical vein endothelial cells [22].

In this study, we examined the inhibitory effects of 3AOA on VEGF-A-induced lymphangiogenesis through in vitro experimentations using SCCVII cells and human lymphatic microvascular endothelial cells (HLMECs). We also investigated the inhibitory effects of 3AOA on VEGF-A-induced lymphangiogenesis and sentinel lymph node metastasis in an oral cancer sentinel lymph node animal model. 3AOA inhibits VEGF-A-induced lymphangiogenesis and sentinel lymph node metastasis in vitro and in vivo via suppression of VEGF-A/VEGFR-1 and VEGFR-2 signaling.

## Methods

### Cell lines and culture

Mouse SCCVII cells were obtained from Dr. Han-Sin Jeong (Samsung Medical Center, Seoul, Korea) and maintained in RPMI-1640 medium (HyClone, Logan, UT) containing 10% (*v*/v) fetal bovine serum (FBS; HyClone) in a 5% CO_2_ humidified incubator at 37 °C. HLMECs (Cat No. CC-2812, Lonza, Basel, Switzerland) were maintained in EGM-2 MV bullet kit medium (Lonza) containing 20% FBS in a humidified 5% CO_2_ incubator at 37 °C.

### Animals and the oral cancer sentinel lymph node animal model

BALB/c 5 week old female mice were purchased from ORIENT BIO Inc. (Seongnam, Korea). Mice received water and food ad libitum while quarantined in a pathogen free environment with a 12 h light and 12 h dark photoperiod in an animal care facility approved by the Institutional Animal Care and Use Committee of Kyung Hee University. Animal care and experimental methods followed the guidelines of Kyung Hee University for care and use of laboratory animals.

To establish an oral cancer sentinel lymph node animal model, SCCVII and mouse VEGF-A overexpressing SCCVII cells (5 × 10^5^ cells/50 μL PBS) were injected submucosally into the right border of the tongue of BALB/c mice. Mice were randomly divided into groups of seven mice each. Each group was treated with an intraperitoneal injection of either 3AOA (1 mg/kg in PBS) or PBS every 2 days for 14 days. All mice were monitored daily for 14 days. The lymph node to which the tumor cell is first drained from the primary tumor is called the sentinel lymph node. To detect the sentinel lymph node in our animal model, we used the blue dye (Evan’s Blue dye) injection method, which is one of the methods used for an actual sentinel lymph node biopsy. One hour after Evan’s blue dye was injected around the primary tumor, mice were sacrificed with the method of euthanasia using CO_2_ inhalation. The blue stained lymph node (among the lymph nodes near the primary tumor) was defined as the sentinel lymph node, distinguishing it from other lymph nodes and excised.

### Tissue preparation

Sentinel lymph nodes and primary tumors were excised 14 days after tumor cell injection from mice in each group. On the terminal day, the volumes of sentinel lymph nodes and primary tongue tumors were measured. The length and width of sentinel lymph nodes and tumors were measured using a caliper, and the standard formula [width squared × length × 0.5] was used to calculate the tumor volume. Sentinel lymph nodes and tumors were immediately fixed with 10% neutral buffered formalin overnight, and then embedded in paraffin. Paraffin embedded tissues were sectioned to a 5 μm thickness. The sentinel lymph node metastasis rates were confirmed using H & E staining and cytokeratin immunohistochemical analysis of the sentinel lymph node paraffin sections. The paraffin sections were also used for LYVE-1 immunohistochemial analysis for detection of lymphangiogenesis.

### RT-PCR analysis

Total RNA was isolated from SCCVII cells using Trizol reagent (Invitrogen, Carlsbad, CA) according to the protocol supplied by the manufacturer. Two μg of total RNA was used for cDNA synthesis with an Improm-II Reverse Transcription System kit (Promega, Madison, WI). The reverse transcription procedure was performed following the manufacturer-provided protocol in a 20 μL reaction mixtures containing oligo(dT) primer. PCR products were obtained from Dream taq (Thermo Fisher Scientific Inc., MA, USA), and 2 μL of cDNA was used for PCR with specific primers using mouse VEGF-A, 5’-GCCCTGAGTCAAGAGGACAG-3′ (forward) and 5′-GAAGGGAAGATGAGGAAGGG-3′ (reverse); mo-use VEGF-B, 5’-GACATCATCCATCCCACTCC-3′ (forward) and 5’-CTCACTTGACCAGGGTGGTT-3′ (reverse); mouse VEGF-C, 5’-CCACAGTGTCAGGCAGCTAA-3′ (forward) and 5’-ACTGCATGTTTGATGGTGGA-3′ (reverse); and finally mouse VEGF-D, 5’-GTATGGACTCACGCTCAGCA-3′ (forward) and 5’-TTTGGTGTTATCCCACAGCA-3′ (reverse). PCR products were resolved on 1% agarose/Tris-acetate EDTA gels that were electrophoresed then visualized with ethidium bromide. PCR product band intensity values were determined using the Image J program (NIH, MD, USA).

### Protein extraction, western blot analysis, and immunoprecipitation

Cells were washed with PBS and lyzed with RIPA buffer (Pierce, Rockford, IL) supplemented with a protease inhibitor cocktail (Sigma-Aldrich, St. Louis, MO) and a phosphatase inhibitor cocktail (Sigma-Aldrich). Protein extracts were collected via centrifugation at 15,000×g for 10 min. Protein concentrations were determined using an RC/DC protein assay reagent (Bio-Rad, Hercules, CA). Protein extracts were separated using 6 and 10% SDS-PAGE and transferred onto PVDF membranes (PALL, USA). Membranes were pre-incubated in a blocking solution [3% skim milk in TBS including 0.1% Tween-20] for 1 h and incubated with anti-VEGF-A, anti-VEGFR-1, and anti-VEGFR-2 primary antibodies at 1:1000 dilution in a blocking solution (Santa Cruz Biotech. Inc., Santa Cruz, CA) or anti-phospho FAK, anti-phospho ERK1/2, anti-phospho PI3K, and anti-phospho AKT antibodies at a 1:2000 dilution in a blocking solution (Santa Cruz Biotech. Inc.) overnight at 4 °C, and probed with peroxidase conjugated anti-rabbit IgG, anti-goat IgG, and anti-mouse IgG antibodies at a 1:5000 dilution in a blocking solution (Sigma-Aldrich). Protein bands were detected using enhanced chemiluminescent Western blotting detection reagent (Thermo Fisher Scientific Inc.). Protein extracts were also immunoprecipitated using a mouse anti-phospho-tyr antibody (Santa Cruz Biotech. Inc.) and an ImmunoCruz™ IP/WB Optima kit (Santa Cruz Biotech. Inc.). Immunoprecipitated proteins were subjected to SDS-PAGE (6%) and Western blotting using mouse anti-VEGFR-1 and goat anti-VEGFR-2 antibodies (Santa Cruz Biotech. Inc.).

### ELISA assay

SCCVII cells were treated with 2.5 and 5 μM 3AOA in a serum free medium containing 100 μM CoCl_2_. The conditioned medium was collected, and 100 μL of conditioned medium was incubated for 2 h at room temperature in a microwell plate coated with anti-VEGF-A monoclonal antibody (R&D System Inc., Minneapolis, MN). After three washes, a horseradish peroxidase conjugated polyclonal VEGF antibody (Santa Cruz Biotech. Inc.) was added, followed by additional incubation for 2 h at room temperature. After addition of a color reagent, the absorbance was measured at 450 nm in an EL800 Universal Microplate Reader (Biotek Instruments Inc.)

### HLMEC proliferation assay

HLMECs (5 × 10^4^ cells) in EBM-2 containing 1% FBS were added to each well of a gelatin coated 24-well plates. After addition of 20 ng/mL rhVEGF-A and/or 3AOA (2.5, 5 μM), cells were incubated for 48 h. Cells were then trypsinized and counted using a hemocytometer. Cell density values obtained based on three independent experiments were represented as bar diagrams.

### HLMEC tube formation assay

One hundred fifty μL of a 1:1 mixture of EBM-2 and growth factor reduced Matrigel (Corning, MA, USA) was added to each well of the 48-well plate and let to polymerize at 37°C for 12 h. HLMECs (5 × 10^4^ cells) in 0.5 mL of EBM-2 containing 1% (*v*/v) FBS, 20 ng/mL rhVEGF-A, and/or 3AOA (2.5, 5 μM) were added to each well. After 8 h, cells were photographed under a inverted phase contrast microscope using a digital single-lens reflex camera and total tube lengths of a unit area were quantified using the Image J program (NIH).

### HLMEC migration assay

Migration assay of HLMEC was performed using 24-well and transwell inserts with 8.0 μm pore sized polycarbonate membrane (SPL Life Science, Korea). Polycarbonate membranes of the transwell inserts were coated with 0.1% (*w*/*v*) gelatin in PBS for 1 h at 37°C. HLMECs (5 × 10^4^ cells) in EBM-2 containing 1% FBS with 2.5 and 5 μM 3AOA were added to the upper compartment of the transwell insert. EBM-2 containing 1% (*v*/v) FBS and 20 ng/ml rhVEGF-A was added to the lower compartment to stimulate cell migration. After a 24 h incubation at 37°C, cells on the top surface of membranes were wiped off with cotton balls, and cells migrated to the underside of membrane were fixed with methanol, stained with a hematoxylin solution (Sigma-Aldrich). Five different digital images per well were obtained, and the migrated cells of a unit area were counted. Each sample was assayed twice and the experiment was repeated twice.

### In vivo Matrigel plug assay

Three hundred μL Matrigel containing 500 ng/mL rhVEGF-A and/or 5 μM 3AOA were injected bilaterally into the flank areas of 5-week old female BALB/c mice (Orient Bio Inc.). After 14 days of injection, Matrigel plugs were excised and fixed in 10% neutral buffered formalin before immunohistochemical analysis.

### Immunohistochemistry

Tongues (primary tumors), sentinel lymph nodes, and Matrigel plugs were immediately exiced from the sacrificed mice and fixed for immunohistological examination. Tongues (primary tumors), sentinel lymph nodes, and Matrigel plugs were fixed overnight in 10% neutral buffered formalin and then embedded in paraffin. Paraffin-embedded tissues were sectioned to a thickness of 5 μm. Paraffin sections were deparaffinized in xylene, rehydrated in sequentially diluted ethanol, and washed with distilled water. After that, sections were boiled in a 10 mM sodium citrate (pH 6.0) for 10 min. To inhibit the activity of endogenous peroxidase, sections were incubated with methanol containig 1% hydrogen peroxide for 10 min, then blocked with 10% normal serum (Vector Laboratories, Burlingame, CA) for 1 h, followed by incubation overnight in anti-Cytokeratin and anti-LYVE-1 (Abcam, Cambridge, UK) primary antibodies diluted with the blocking solution. Sections were probed with horseradish peroxidase conjugated anti-rabbit IgG antibody, and incubated with DAB solution (Vector Laboratories) until the desired stain intensity developed. After counterstaining with hematoxylin, the sections were examined under the Olympus BX21 inverted microscope (Olympus, Japan). To analyze immunohistochemical signals within specimens, all sections were digitized under 200× objective magnification and images were captured. And analyzed using the Image J program.

### Statistical analysis

All data are presented as a mean ± S.D. or S.E.. Student’s *t*-test was used to compare VEGF-A-treated groups with PBS-treated control groups, and compare 3AOA-treated groups with VEGF-A-treated groups. (^*^p < 0.05, ^**^p < 0.01, ^***^ *p* < 0.001).

## Results

### Effects of 3-*O*-acetyloleanolic acid (3AOA) on tumor growth and lymph node metastasis in an oral cancer sentinel lymph node animal model

**Fig. 1:**
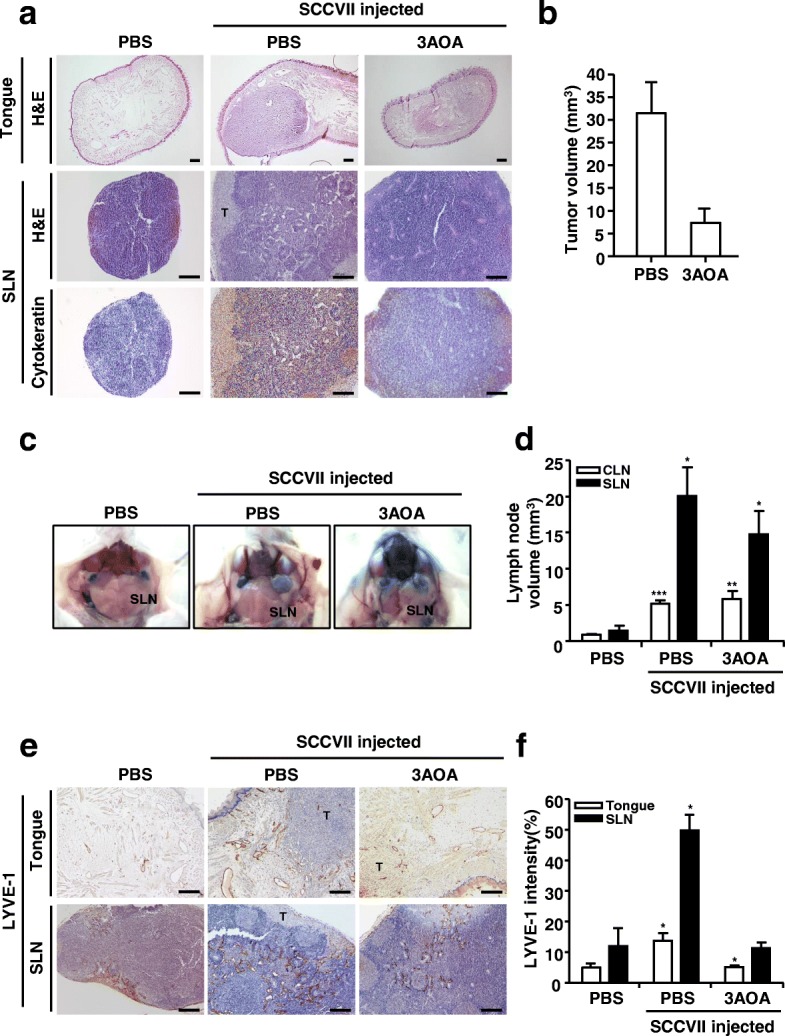
Effects of 3AOA on tumor growth and lymphangiogenesis in an oral cancer sentinel lymph node animal model. **a**, Tumor growth and metastasis to sentinel lymph node **b**, Tumor volume **c**, Image of sentinel lymph nodes **d**, Sentinel lymph node volume **e-f**, Lymphatic vessels in tumor and sentinel lymph node sections. Tumor growth of each group was confirmed by hematoxylin and eosin staining of tumor(tongue) sections. And metastasis to sentinel lymph node was confirmed using hematoxylin and eosin staining, and immunohistochemical analysis with anti-cytokeratin antibody of sentinel lymph node sections. Tumor sections were digitized and microscopic images were captured under a 100× objective magnification. Scale bar = 200 μm. Sentinel lymph node sections were digitized and microscopic images were captured under a 200× objective magnification. Scale bar = 200 μm. Lymphatic vessels in tumor and sentinel lymph node sections were determined using the immunohistochemical analysis with anti-LYVE-1. All sections were digitized and microscopic images were captured under a 200× objective magnification. Scale bar = 200 μm. Immunohistochemical intensity values of LYVE-1 from captured images were analyzed by the Image J program and represented as a bar diagrams. Data are presented as a mean ± S.D. (^*^p < 0.05, ^**^p < 0.01, ^***^p < 0.001). T = tumor; SLN = Sentinel lymph node

To confirm the effects of the angiogenesis inhibitor 3AOA on oral cancer lymph node metastasis, we established an oral cancer sentinel lymph node (OCSLN) animal model (Fig. 1a-f). Firstly, SCCVII cells (5 × 10^5^ cells/50 μL) were injected into the right submucosa of the mouse tongue. After 2 weeks, we confirmed tumor formation at the tumor cell injection site. One hour after peritumoral injection of Evans blue dye, tumor cells drained to the sentinel lymph node. We observed microenvironmental changes and enlargement of sentinel lymph nodes before tumor cell metastasis in SCCVII injected mice (Fig. 1c-d) and tumor cell metastasis to the sentinel lymph node (Fig. 1a).

Inhibitory effects of 3AOA against sentinel lymph node metastasis in the OCSLN animal model were observed. In the SCCVII and 1 mg/kg 3AOA injected groups, growth and metastasis of primary tumors were inhibited, compared with the control group (SCCVII only injected group) (Fig. 1a-b). In the SCCVII and 1 mg/kg 3AOA injected groups, enlargement of sentinel lymph nodes was also inhibited, compared with the control group (Fig. 1c-d). We confirmed the effects of 3AOA on tumor-related lymphangiogenesis, the essential course of lymph node metastasis through immunohistochemical analysis of primary tumor and sentinel lymph node tissues using the lymphatic vessel marker LYVE-1 antibody. In both primary tumors and sentinel lymph nodes, lymphangiogenesis was stimulated by tumor cells in the control group (SCCVII only injected group), but stimulated lymphangiogenesis was inhibited by 3AOA treatment (Fig. 1e-f). Thus, 3AOA inhibits tumor growth, tumor-induced lymphangiogenesis, and lymph node metastasis in an OCSLN animal model.

### Effects of 3-*O*-acetyloleanolic acid on expression of lymphangiogenic factors in CoCl_2_-treated SCCVII cells

**Fig. 2:**
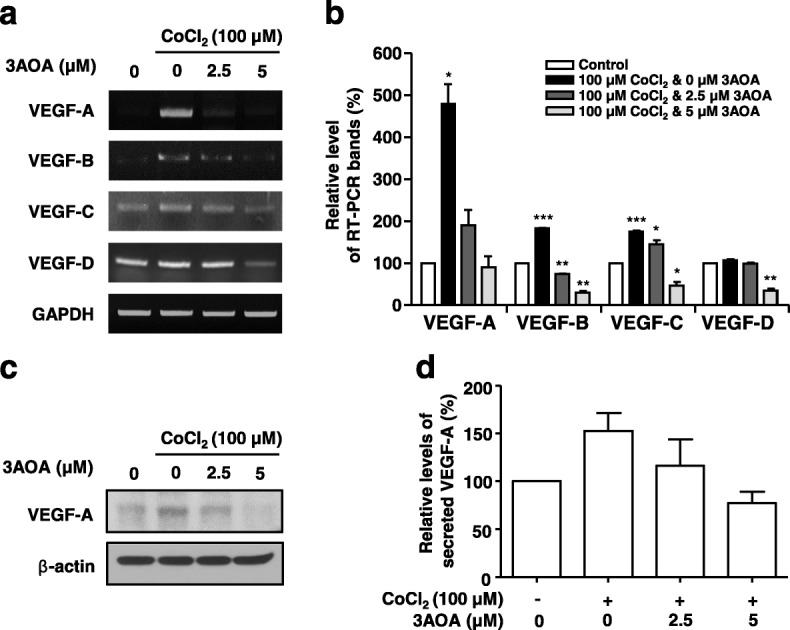
Effects of 3AOA on the expression of VEGF family proteins in SCCVII cells treated with CoCl_2_ . **a**, cDNAs were generated from total RNAs treated with DNase I, and PCR reaction was performed with specific primers of VEGF-A, -B, -C, −D and GAPDH. **b**, PCR products from three independent experiments (**a**) were quantified and represented as a bar diagram. The levels of the VEGF-A, -B, -C, and -D transcripts in the control (3AOA- and CoCl_2_-untreated cells) were estimated as 100%. **c**, The protein level of VEGF-A in the intracellular fraction was determined using western blot with anti-VEGF-A antibody. **d**, The secreted VEGF-A protein level in a conditioned medium was determined using an ELISA assay. The amounts of VEGF-A obtained in three independent experiments were quantified and represented as a bar diagram. The level of VEGF-A in the conditioned medium of the control was estimated as 100%. Data are presented as a mean ± S.D. of three independent experiments (^*^p < 0.05, ^**^p < 0.01, ^***^p < 0.001)

To investigate the effects of 3AOA on tumor-related lymphangiogenesis, we confirmed expression of VEGF family lymphangiogenic factors in the SCCVII cells treated with CoCl_2_ using RT-PCR and Western blot analysis. Total RNA was prepared from the SCCVII cells treated with CoCl_2_ in the presence and absence of 3AOA. β-actin was used as an internal control. The VEGF-A mRNA transcript level was increased by 379.8% in CoCl_2_-treated SCCVII cells, compared with CoCl_2_-untreated SCCVII cells (Fig. 2a-b). The increased level of the VEGF-A transcript ater CoCl_2_ treatment was reduced by 76.3% in 2.5 μM and by 102.5% in 5 μM 3AOA-treated cells. The VEGF-C mRNA transcript level also increased 82.5% in CoCl_2_-treated SCCVII cells, compared with CoCl_2_-untreated SCCVII cells. The increased level of the VEGF-C mRNA transcript after CoCl_2_ treatment was reduced by 40.4% in 2.5 μM and by 144.2% in 5 μM 3AOA-treated cells, respectively. Expression of the VEGF-A protein was further confirmed using Western blot analysis (Fig. 2c). The VEGF-A protein level in CoCl_2_-treated SCCVII cells was increased, compared with the level in CoCl_2_-untreated SCCVII cells. The increased VEGF-A protein level was reduced in 2.5 μM and 5 μM 3AOA-treated cells dose-dependently (Fig. 2c). Additionally, we confirmed the level of the secreted VEGF-A protein in a conditioned medium using an ELISA assay. The secreted VEGF-A protein level in the medium of CoCl_2_-treated SCCVII cells was increased by 52.8%, compared with the level in the medium of CoCl_2_-untreated SCCVII cells. The increased VEGF-A protein levels in the conditioned medium of CoCl_2_-treated SCCVII cells were reduced by 69.1 and 142.9%, respectively, due to 2.5 μM and 5 μM 3AOA treatments (Fig. 2d). Thus, VEGF-A is the most significantly increased lymphangiogenic factor in hypoxic SCCVII cells that are induced by CoCl_2_ and increased expression of VEGF-A is reduced by 3AOA.

### Effect of 3-*O*-acetyloleanolic acid on VEGF-A-induced proliferation, tube formation, and migration of HLMECs

**Fig. 3:**
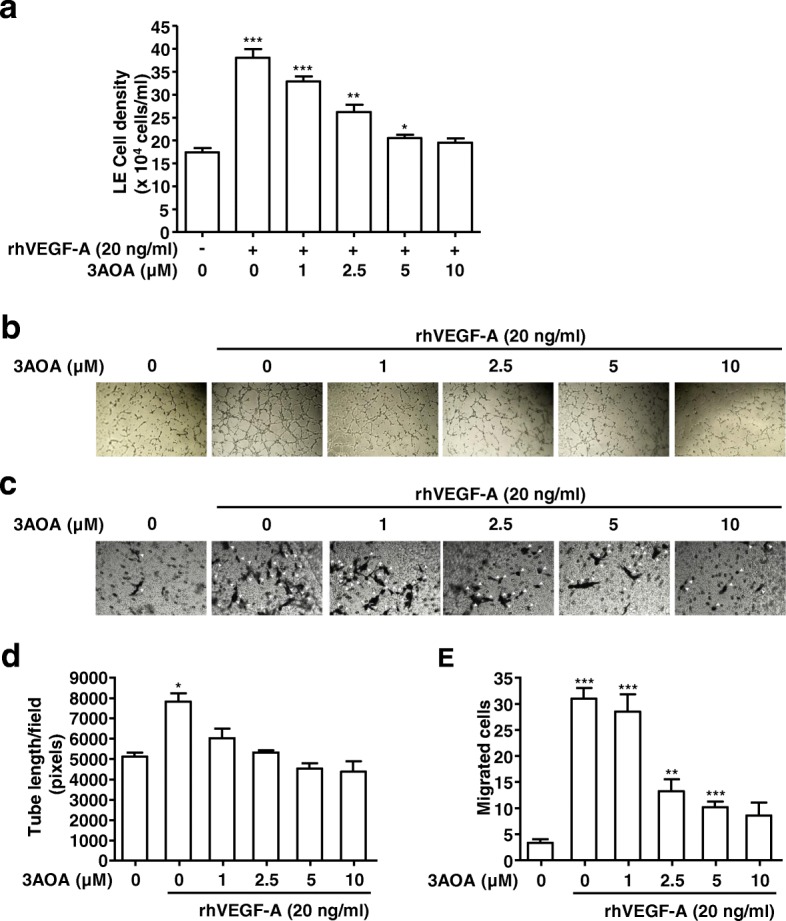
Effects of 3AOA on proliferation, tube formation, and migration in HLMECs stimulated with rhVEGF-A. **a**, Proliferation in HLMECs stimulated rhVEGF-A. Cells were detached and counted using a hemocytometer. **b** and **d**, Tube formation in HLMECs stimulated rhVEGF-A. Cells were imaged under a inverted phase contrast microscope using a digital single-lens reflex camera. Total tube lengths of a unit area were calculated using the Image J program. **c** and **e**, The migrated cells to the underside of membranes were fixed with methanol, stained with hematoxylin solution, and then imaged under a inverted phase contrast microscope using a digital camera. Five digital images per well for (C) were obtained, and the numbers of migrated HLMECs were counted. Each sample was assayed in duplicate. Numbers of migrated HLMECs present in 320 mm^2^ are presented as a bar diagram. Data are presented as a mean ± S.D. of three independent experiments (^*^p < 0.05, ^**^p < 0.01, ^***^p < 0.001)

To confirm the effects of 3AOA on VEGF-A-induced lymphangiogenesis in vitro, we performed proliferation, tube formation, and migration assays in rhVEGF-A-treated HLMECs. HLMECs treated with rhVEGF-A (20 ng/ml) in the presence and absence of different concentrations of 3AOA (1, 2.5, 5, 10 μM) for 48 h. Subsequent to assay, cell density values were measured after trypsinization using a hemocytometer (Fig. 3a). Proliferation of HLMECs was stimulated by 118.7% due to rhVEGF-A, compared with rhVEGF-A-untreated HLMECs. However, cell density values of HMLECs treated with rhVEGF-A (20 ng/ml) and different concentrations of 3AOA (1, 2.5, 5, 10 μM) were decreased dose-dependently.

The effect of 3AOA on the HLMEC tube formation that was induced by rhVEGF-A was investigated using Matrigel-precoated 48-well plates. HLMECs were treated with rhVEGF-A (20 ng/ml) in the presence and absence of different concentrations of 3AOA (1, 2.5, 5, 10 μM) for 8 h in Matrigel-precoated 48-well plates. After 8 h, HLMEC tube formation was increased by 53%, compared with rhVEGF-A-untreated HLMECs. However, increased HLMEC tube formation due to rhVEGF-A was decreased by 66.8, 92.8, 121.7, and 127.4% in the presence of 1, 2.5, 5, and 10 μM 3AOA, respectively (Fig. 3b and d).

The effect of 3AOA on the migration of HLMECs that was induced by rhVEGF-A was determined using 24-well and transwell inserts with 8.0 μm pore size polycarbonate membrane (Fig. 3c and e). A medium containing 20 ng/mL rhVEGF-A was added to the bottom chamber of a transwell plate. After 24 h incubation, the number of HLMECs that migrated to underside of the polycarbonate membranes was increased by 811.8%. HLMEC migration that was stimulated by rhVEGF-A was dramatically reduced by 3AOA. Levels of 1, 2.5, 5, and 10 μM 3AOA reduced the stimulated migration of HLMEC by 9, 64.3, 78.5, and 80.3%, respectively (Fig. 3c and e). Thus 3AOA inhibits proliferation, tube formation, and migration in rhVEGF-A-stimulated HLMECs.

### Effects of 3-*O*-acetyloleanolic acid on expression of VEGFR-1 and VEGFR-2 and activation of VEGFR-1, VEGFR-2, and lymphangiogenesis related downstream signaling factors in rhVEGF-A-treated HLMECs

**Fig. 4:**
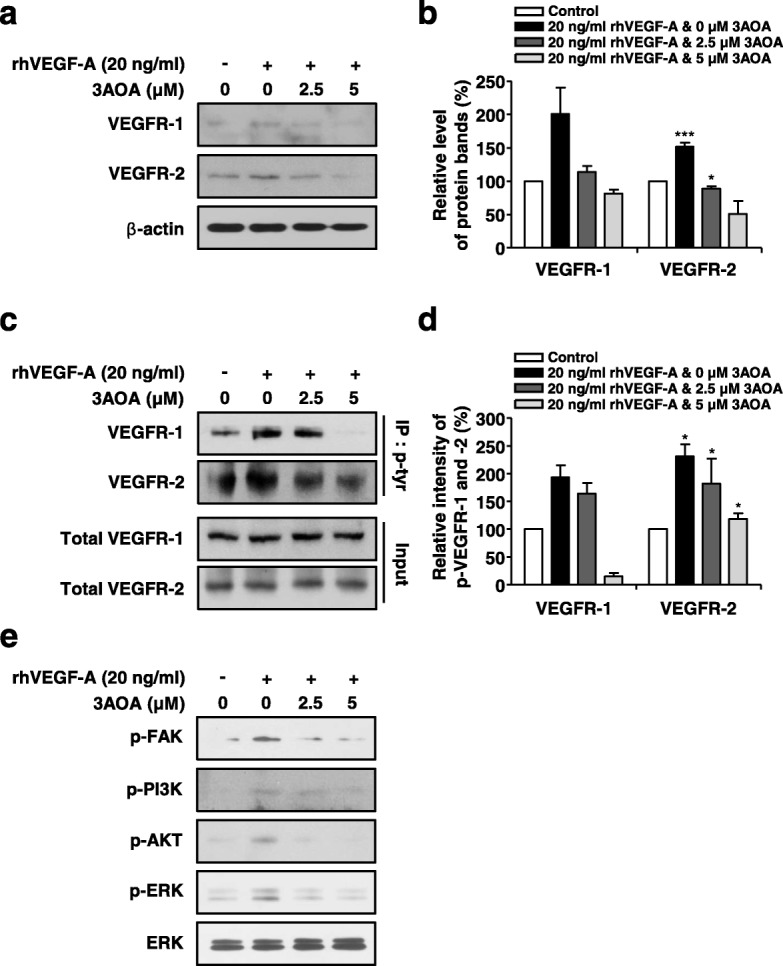
Effects of 3AOA on expressions of VEGFR-1 and VEGFR-2, and activation of VEGFR-1, VEGFR-2 and lymphangiogenesis related downstream signaling factors in rhVEGF-A-treated HLMECs. **a-b** Expression levels of VEGFR-1 and -2 proteins were determined using Western blot analysis. Amounts of VEGFR-1 and -2 obtained in three independent experiments were quantified and represented as a bar diagram. Levels of VEGFR-1 and -2 in 3AOA- and rhVEGF-A-untreated cells were estimated as 100%. **c-d**, Cell lysates were immunoprecipitated with anti-phospho-Tyr (anti-p-Tyr). The level of phosphorylated VEGFR-1 and -2 in immunoprecipitates was detected using Western blot analysis with anti-VEGFR-1 and anti-VEGFR-2. Phosphorylation levels of VEGFR-1 and -2 obtained in three independent experiments were quantified and represented as a bar diagram. Phosphorylation levels of VEGFR-1 and -2 in 3AOA- and rhVEGF-A-untreated cells were estimated as 100%. **e**, HLMECs were serum starved for 6 h, then were treated with different concentrations of 3AOA (0, 2.5, 5 μM) in the presence of rhVEGF-A (20 ng/mL) for 60 min. The phosphorylation levels of FAK, PI3K, AKT, and ERK1/2 were determined using Western blot analysis with anti-p-FAK, anti-p-PI3K, anti-p-AKT, and anti-p-ERK1/2. Data are presented as a mean ± S.D. of three independent experiments (^*^p < 0.05, ^**^p < 0.01, ^***^p < 0.001)

Expression levels of VEGFR-1 and VEGFR-2, the cell surface receptor of VEGF-A in rhVEGF-A treated HLMECs were determined using Western blot analysis. Expressions of VEGFR-1 and VEGFR-2 proteins were increased by 100.9 and 51.8%, respectively, in rhVEGF-A treated HLMECs, compared with a rhVEGF-A-untreated control. Expressions of VEGFR-1 and VEGFR-2 that were increased by rhVEGF-A were dramatically reduced by 3AOA treatment. Treatment of 2.5 and 5 μM 3AOA reduced VEGFR-1 expression levels by 86.4 and 118.2%, respectively, compared with rhVEGF-A treated cells. In addition, 2.5 and 5 μM 3AOA treatments reduced VEGFR-2 expression levels by 121.4 and 194.8%, respectively, compared with rhVEGF-A treated cells (Fig. 4a-b). Thus, 3AOA inhibits the expressions of VEGFR-1 and VEGFR-2 that are induced by rhVEGF-A in HLMECs.

To investigate the effects of 3AOA on activation of VEGFR-1 and VEGFR-2, immunoprecipitation analysis using the anti-phospho-tyr antibody and Western blot analysis using the anti-VEGFR-1 and anti-VEGFR-2 antibodies were performed. rhVEGF-A-treated HLMEC lysates were immunoprecipitated using the anti-phospho-tyr antibody and Western blot analysis using the anti-VEGFR-1 and anti-VEGFR-2 antibodies confirmed phosphorylation levels of VEGFR-1 and VEGFR-2 in immunoprecipitated proteins. Phosphorylated VEGFR-1 and VEGFR-2 levels in rhVEGF-A-treated HLMECs increased by 93.9 and 131.6%, compared with rhVEGF-A-untreated HLMECs. Phosphorylated VEGFR-1 levels in rhVEGF-A and 3AOA (2.5 or 5 μM) treated HLMECs were reduced by 31.1 and 190.6%, respectively, compared with rhVEGF-A only treated HLMECs. Phosphorylated VEGFR-2 levels in rhVEGF-A and 3AOA (2.5 or 5 μM) treated HLMECs were reduced by 37.3 and 86.3%, respectively, compared with rhVEGF-A only treated HLMECs (Fig. 4c-d).

Binding of VEGF-A and VEGFR-1 and/or VEGFR-2 promotes lymphatic endothelial cell proliferation and migration via the PI3K/AKT and ERK pathway. To confirm whether 3AOA modulates this pathway activated by VEGF-A, we performed Western blot analysis using anti-phospho FAK, anti-phospho PI3K, anti-phospho AKT, and anti-phospho ERK antibodies. Both 2.5 μM and 5 μM levels of 3AOA inhibited FAK, PI3K, AKT, and ERK phosphorylation in rhVEGF-A-treated HLMECs (Fig. 4e). Thus, 3AOA probably reduces the expressions of VEGFR-1 and VEGFR-2 that are increased by rhVEGF-A, and 3AOA probably inhibits the activation of VEGFR-1, VEGFR-2, and lymphangiogenesis related downstream signaling factors that are stimulated by rhVEGF-A.

### Effects of 3-*O*-acetyloleanolic acid on rhVEGF-A-induced formation of lymphatic vessels in vivo in a Matrigel plug

**Fig. 5:**
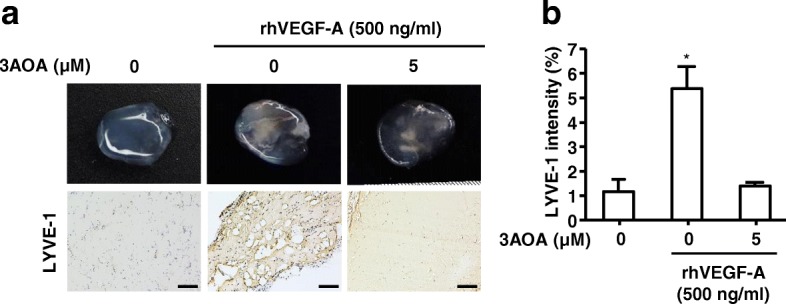
Effects of 3AOA on VEGF-A-induced lymphatic vessel formation in an in vivo Matrigel plug. **a**, Matrigel plugs were excised and photographed using a digital camera. The lymphatic vessel density values in Matrigel plug sections were measured using the immunohistochemical analysis with anti-LYVE-1 antibody. All Matrigel sections were digitalized and microscopic images were captured under 200× objective magnification. Scale bar = 200 μm. **b**, Immunohistochemical intensity values (LYVE-1) from captured images were analyzed by the Image J program and represented as a bar diagram. Data are presented as a mean ± S.D. (^*^p < 0.05)

To determine the effect of 3AOA on rhVEGF-A-induced lymphangiogenesis in vivo, we performed a Matrigel plug assay using BALB/c mice. Matrigel plugs containing 500 ng/mL rhVEGF-A and/or 5 μM 3AOA were injected bilaterally into the mouse flank. After 14 days, Matrigel plugs were excised and imaged and rhVEGF-A-treated Matrigel plugs showed capillary vessels inside the plugs. However capillary vessel formation in plugs was inhibited by 3AOA (Fig. 5a). To confirm the lymphatic vessel density in excised plugs, we performed immunohistochemical analysis using the antibody against LYVE-1, which is a lymphatic vessel marker. The lymphatic vessel density shown by anti-LYVE-1 staining was notably increased in rhVEGF-A-treated Matrigel plugs, compared with rhVEGF-A-untreated Matrigel plugs. The staining intensity of LYVE-1 in 3AOA-treated Matrigel plugs was decreased to 74%, compared with rhVEGF-A-treated Matrigel plugs (Fig. 5a-b). Thus, rhVEGF-A stimulates lymphatic vessel formation in vivo, and 3AOA inhibits rhVEGF-A-induced lymphatic vessel formation.

### Effects of 3-*O*-acetyloleanolic acid on lymphangiogenesis, and lymph node metastasis in a VEGF-A-induced OCSLN animal model

**Fig. 6:**
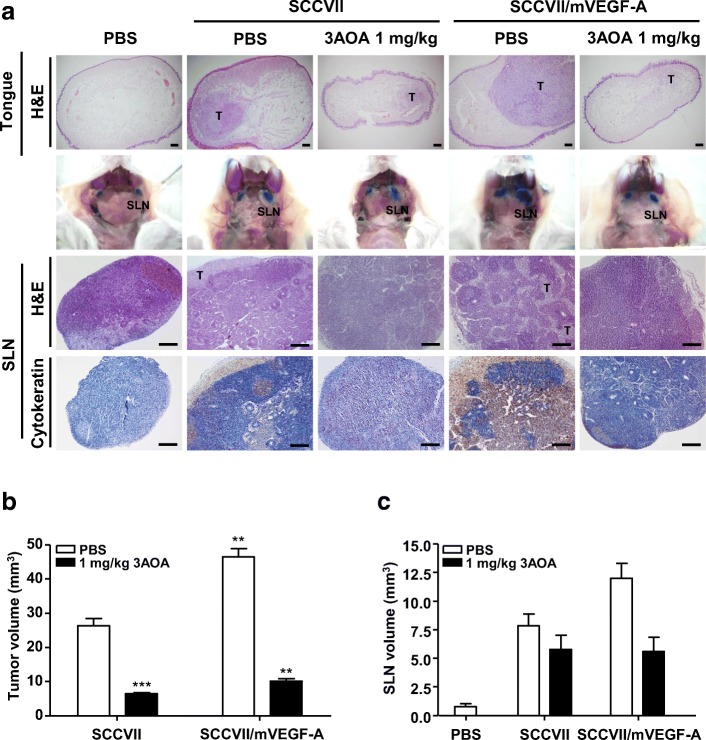
Effects of 3AOA on tumor growth, and sentinel lymph node enlargement and lymph node metastasis in a VEGF-A-induced oral cancer sentinel lymph node animal model. **a**, Tumor (tongue) and SLN sections of mice of each group were analyzed using hematoxylin and eosin staining, and immunohistochemical analysis with anti-cytokeratin antibody. All tumor sections were digitized, and microscopic images were captured under a 100× objective magnification. Scale bar = 200 μm. All SLN sections were digitized and images were captured under 200× objective magnification. Scale bar = 200 μm. **b-c**, Tumor volume and SLN volume were measured using a caliper. Data are presented as a mean ± S.D. (^**^p < 0.01, ^***^p < 0.001). T = tumor; SLN = Sentinel lymph node

To confirm the in vivo effects of 3AOA on VEGF-A-induced lymphangiogenesis and lymph node metastasis, we established the a mouse VEGF-A overexpressing SCCVII cell (SCCVII/mVEGF-A) and VEGF-A-induced OCSLN animal model using cells. To establish mVEGF-A overexpression in SCCVII cells, we constructed pCMV-Taq2C/FLAG-mVEGF-A plasmid DNA and transfected the plasmid DNA into SCCVII cells. mVEGF-A overexpression in transfected cells was confirmed by RT-PCR and Western blot analysis (Additional file 1). In a control group (normal SCCVII injected group) the primary tumor volume was 26.3 ± 2.1 mm^3^, and the primary tumor volume of the SCCVII/mVEGF-A injected group was 46.47 ± 2.4 mm^3^. Tumor growth was stimulated by 76.7% in the SCCVII/mVEGF-A injected group, compared with SCCVII injected group. In the SCCVII/mVEGF-A and 1 mg/kg 3AOA injected group, the primary tumor volume was 10.15 ± 1.1 mm^3^. Tumor growth stimulated by mVEGF-A overexpression was inhibited by 180.1% in the SCCVII/mVEGF-A and 1 mg/kg 3AOA injected group (Fig. 6a-b). Also, enlargement of sentinel lymph node in the SCCVII/mVEGF-A injected group was stimulated, compared with normal SCCVII injected group from 7.84 ± 1.02 mm^3^ to 11.9 ± 1.3 mm^3^. However, the stimulated enlargement of sentinel lymph nodes in the SCCVII/mVEGF-A injected group was inhibited by 5.59 ± 1.24 mm^3^ due to 3AOA (Fig. 6a and c).

**Fig. 7:**
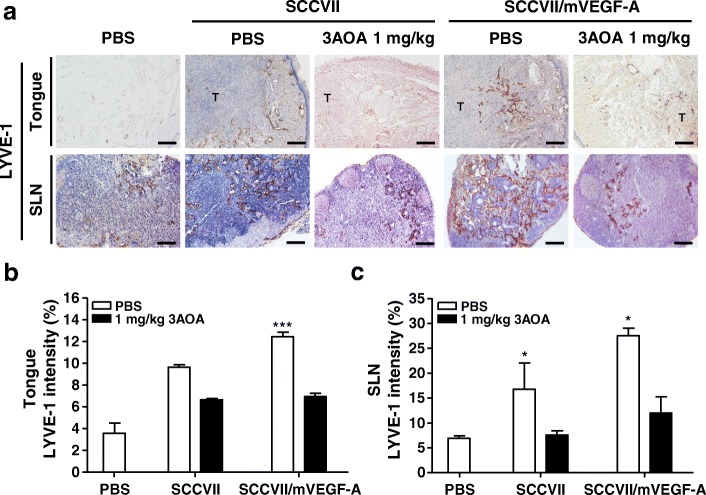
Effect of 3AOA on tumor-induced lymphangiogenesis in a VEGF-A-induced oral cancer sentinel lymph node animal model. **a**, Lymphatic vessel density values in tumor and SLN sections were measured by immunohistochemical analysis using anti-LYVE-1 antibody. All sections were digitalized and images were captured under 200× objective magnification. Scale bar = 200 μm. **b-c**, Immunohistochemical intensity values of LYVE-1 from captured images of tumors and SLN were analyzed via the Image J program and represented as a bar diagram. Data are presented as a mean ± S.D. (^*^p < 0.05, ^***^p < 0.001). T = tumor; SLN = Sentinel lymph node

We investigated the effects of 3AOA on VEGF-A-induced lymphangiogenesis in primary tumor and sentinel lymph nodes via immunohistochemical analysis using anti-LYVE-1 antibody. LYVE-1 intensity values in primary tumors and sentinel lymph nodes in the SCCVII/mVEGF-A injected group were increased by 29 and 64.2%, respectively, compare with control group (normal SCCVII injected group). Increased LYVE-1 density values in the SCCVII/mVEGF-A injected group decreased by 56.8 and 92.8% due to 3AOA, compared with SCCVII/mVEGF-A injected group, respectively (Fig. 7). To determine the presence of metastasis and the extent of spread within the sentinel lymph node, we performed H & E and cytokeratin staining of the sentinel lymph node tissues of each group. In 3AOA treated group, we observed a notable decrease in the rate of sentinel lymph node metastasis. Metastasis rates were confirmed to be 57.1% (4 of 7 mice) and 71.4% (5 of 7 mice) in the normal SCCVII injected and SCCVII/mVEGF-A injected groups, respectively. The metastasis rate of the SCCVII/mVEGF-A and 1 m/kg 3AOA injected group was 14.3% (1 of 7 mice). Thus, stimulated tumor growth, lymphangiogenesis, and sentinel lymph node metastasis due to VEGF-A in vivo are inhibited by 3AOA.

## Discussion

Oral cancer initially metastasizes sequentially from a primary tumor to regional lymph nodes through the lymphatic system with distant metastases to the lungs, bones, and liver. Lymph node metastasis is a powerful, perhaps the most powerful, prognostic factor for survival of HNSCC patients [23]. The presence or absence of metastasis to sentinel lymph nodes, which is the first draining lymph node, is an especially important factor in determining the treatment method of oral cancer patients. Tumor-induced lymphangiogenesis plays an important role in metastatic spread to lymph nodes via the lymphatic system.

This study investigated the inhibitory effects of 3AOA on tumor-induced lymphangiogenesis and lymph node metastasis using an oral cancer sentinel lymph node animal model. We established an experimental animal model for oral cancer sentinel lymph node metastasis using BALB/c mice and SCCVII cells. A dosage of 1 mg/kg 3AOA for our study was determined based on previous in vivo experiments we conducted using a heterotopic CT-26 colon carcinoma animal model, in which an intraperitoneal injection of 3AOA at a dosage of 1 mg/kg successfully inhibited the tumor growth that was induced by recombinant angiopoietin-1. (data not shown). 3AOA inhibited tumor growth in the animal model (Fig. 1a-b). 3AOA inhibited enlargement of sentinel lymph nodes and migration of cancer cells from primary tumors to sentinel lymph nodes (Fig. 1a and c-d) and 3AOA significantly reduced lymphatic vessel density values in primary tumor and sentinel lymph node specimens (Fig. 1e-f). Thus, 3AOA inhibits tumor-induced lymphangiogenesis and lymph node metastasis.

Tumor-induced lymphangiogenesis is mediated by lymphangiogenic growth factors that are produced and secreted by tumor cells. Lymphangiogenic growth factors, such as the VEGF family, angiopoietin-1, PDGFs, FGF, and other factors, promote metastasis of tumor cells to lymph nodes [24]. Therefore, we investigated the effect of 3AOA on expression of lymphangiogenic growth factors in cells treated with CoCl_2_. CoCl_2_, a chemical hypoxia-mimicking agent that allows preferential growth of hypoxic cancer cells over control cells, leading to a higher cell number [25]. CoCl_2_ prevents proteasomal degradation of hypoxia-inducible factor-1α (HIF-1α), resulting in a hypoxia-like condition such as might be produced by an in vivo tumor. VEGF-A expression increased in endothelial and cancer cells under the hypoxic conditions induced by HIF-1α stabilization [26]. Our preliminary study indicated that under hypoxia conditions, the changes in expression of VEGF-A was much greater than changes in expressions of VEGF-C or VEGF-D [17]. Increased expression of VEGF-A due to CoCl_2_ was greatly reduced by 3AOA with little or no change in expressions of VEGF-B, -C, −D (Fig. 2), and other lymphangiogenic growth factors (data not shown). Thus, VEGF-A is one of major lymphangiogenic factors in SCCVII cells responding to 3AOA in the hypoxia condition.

In general, VEGF-A is known as an angiogenic factor that mediates angiogenesis by binding to its cell surface receptors VEGFR-1 and VEGFR-2 in endothelial cells of the vascular system [16, 27]. However, several recent studies have reported that VEGF-A promotes proliferation and migration of lymphatic endothelial cells [12, 28] and VEGF-A overexpression induces tumor and sentinel lymph node lymphangiogenesis and lymphatic metastasis [11].

We investigated the anti-lymphangiogenic effect of 3A-OA on VEGF-A-induced lymphangiogenesis both in vitro and in vivo. To investigate the anti-lymphangiogenic effect of 3AOA, concentrations of 2.5 and 5 μM were used for non-cytotoxic levels in SCCVII and HLMEC cells. The in vitro inhibitory effect of 3AOA on VEGF-A-induced lymphangiogenesis was determined based on proliferation, tube formation and migration assays using HLMEC cells. The in vivo inhibitory effect of 3AOA on VEGF-A-induced lymphangiogenesis was determined using a VEGF-A-induced in vivo Matrigel plug assay. The proliferation, tube formation, and migration of HLMECs that were induced by VEGF-A were inhibited by 3AOA (Fig. 3). 3AOA reduced development of new lymphatic vessels in VEGF-A-stimulated Matrigel plugs (Fig. 5). Thus, 3AOA inhibits VEGF-A-induced lymphangiogenesis in vitro and in vivo.

VEGFR-1 and VEGFR-2 exert actions through differing pathways to promote endothelial cell proliferation, migration, and tubular structure formation [27]. In previous experiments, changes in mRNA and protein levels of VEGFR-1, − 2, − 3 were observed after VEGF-A treatment of HLMEC. Although the mRNA and protein levels of VEGFR-3 were induced by VEGF-A treatment, induced levles of mRNA and protein in VEGFR-1 due to VEGF-A treatment were hightest [17]. VEGFR-2 is the predominant mediator for VEGF-A-related responses in endothelial cells. The tyrosine kinase activity of VEGFR-1 is less efficient than the activity of VEGFR-2 and the activation of VEGFR-1 alone is insufficient to induce the proliferative effect of VEGF-A [13]. VEGF-A induces phosphorylation of VEGFR-2 in LECs and stimulates tissue repair-associated lymphangiogenesis [28, 29]. VEGF-A stimulates the expression of VEGFR-2 that is mediated by VEGF-A stimulated activation of ERK1/2 and PI3K/Akt signaling in HUVECs. 3AOA inhibited the expression and phosphorylation of VEGFR-1 and VEGFR-2 that were induced by VEGF-A in HLMECs (Fig. 4a-d), and 3AOA inhibited the phosphorylations of ERK1/2, PI3K, and Akt that were stimulated by VEGF-A in HLMECs (Fig. 4e). Thus, the anti-lymphangiogenic effects of 3AOA are probably mediated via suppression of VEGF-A/VEGFR-1 and VEGFR-2 signaling in HLMECs, and 3AOA probably inhibits VEGF-A-stimulated activation of PI3K, Akt, and ERK1/2 lymphangiogenesis-related signaling factors in HLMEC. Recently, the dietary compound isoliquiritigenin(ISL) has been reported to inhibit neoangiogenesis via the VEGF/VEGFR-2 signaling pathway in breast cancer [30]. ISL does not participate in binding of VEGF and VEGFR-2 in HUVEC cells, but directly binds to the ATP-binding site of the tyrosine kinase domain of VEGFR-2 to inhibit its kinase activation, and also inhibits activation of angiogenesis-related downstream signaling factors. Consequently, ISL can inhibit tumor angiogenesis through the VEGF/VEGFR-2 pathway. Although further studies are needed, we postulate that 3AOA affects VEGFR-2 and inhibits activation of lymphangiogenesis-ralated downstream signaling factors in HLMECs, similar to the made of ISL in HUVECs.

Tumor metastasis to sentinel lymph nodes in VEGF-A transgenic mice occurred more frequently than in wild-type mice. VEGF-A-overexpressing cancer cells maintained their lymphangiogenic activity after metastasis to the sentinel lymph nodes and induced lymphangiogenesis in the sentinel lymph nodes, before tumor cells had metastasized to these tissues [11]. To confirm the effects of 3AOA on VEGF-A-induced tumor-related lymphangiogenesis and tumor metastasis to sentinel lymph nodes in vivo, we established a VEGF-A-induced oral cancer sentinel lymph node animal model using VEGF-A-overexpressing SCCVII cells. In this model, lymphatic vessel density values in primary tumors and sentinel lymph nodes, the sentinel lymph node volume, and the frequency of sentinel lymph node metastasis from primary tumors were increased by VEGF-A. 3AOA inhibited these increases that were due to VEGF-A. Thus, 3AOA inhibits the tumor induced lymphangiogenesis and metastasis to sentinel lymph nodes that is stimulated by VEGF-A.

## Conclusion

In summary, we established the an oral cancer sentinel lymph node animal model using SCCVII cells and BALB/c mice. 3AOA inhibited tumor related lymphangiogenesis and metastasis to sentinel lymph node of tumor cells in the animal model. VEGF-A is a lymphangiogenic factor that is expressed by SCCVII cells under induced hypoxic conditions. 3AOA reduced expression of VEGF-A more than other lymphangiogenic factors in CoCl_2_-treated SCCVII cells. 3AOA inhibited stimulated proliferation, tube formation, and migration in HLMECs that were induced by VEGF-A. In addition, 3AOA inhibited the in vivo formation of lymphatic vessels that was stimulated by VEGF-A in a Matrigel plug. 3AOA suppressed the expression and phosphorylation of VEGFR-1 and -2 that were stimulated by VEGF-A. Also, 3AOA inhibited activation of the lymphangiogenesis-related signaling factors FAK, PI3K, Akt, and ERK1/2. Thus, 3AOA inhibits VEGF-A-induced lymphangiogenesis and sentinel lymph node metastasis in an oral cancer sentinel lymph node animal model. 3AOA inhibited lymphangiogenesis via suppression of the VEGF-A/VEGFR-1 and VEGFR-2 signaling pathways. Thus, 3AOA can be a valuable therapeutic agent for treatment and metastasis prevention in oral cancer.

## Additional file

Additional file 1: Establishment of mVEGF-A overexpressing SCCVII cell. **a**, Schematic representation of the expression plasmid pCMV-Tag 2C/FLAG-mVEGF-A. **b**, Overexpression of mVEGF-A was investigated in non-transfected and stably transfected SCCVII cells using an RT-PCR and Western blot analysis. (PPTX 682 kb)

## Abbreviations

3AOA: 3-*O*-acetyl oleanolic acid
EBM: Endothelial basal medium
EGM: Endothelial growth medium
ERK1/2: Extracellular signaling regulated kinase 1/2
FBS: Fetal bovine serum
FGF: Fibroblast growth factor
HLMEC: Human lymphatic microvascular endothelial cell
LYVE-1: Lymphatic vessel hyaluronan receptor-1
OSCC: Oral squamous cell carcinoma
PI3K: Phosphatidylinositol 3 kinase
rhVEGFR-A: Recombinant human VEGF-A
SCC: Squamous cell carcinoma
VEGF: Vascular endothelial growth factor
VEGFR: Vascular endothelial growth factor receptor
