# Supplementary material for: 3-O-Acetyloleanolic acid inhibits VEGF-A-induced lymphangiogenesis and lymph node metastasis in an oral cancer sentinel lymph node animal model
Source: BMC Cancer. 2018 Jul 5;18:714. doi: 10.1186/s12885-018-4630-0 (PMC6034267; doi:10.1186/s12885-018-4630-0)
Supplement: Supplementary file 1 — Establishment of mVEGF-A overexpressing SCCVII cell. a, Schematic representation of the expression plasmid pCMV-Tag 2C/FLAG-mVEGF-A. b, Overexpression of mVEGF-A was investigated in non-transfected and stably transfected SCCVII cells using an RT-PCR and Western blot analysis. (PPTX 682 kb) [file 12885_2018_4630_MOESM1_ESM.pptx]

## Slide 1
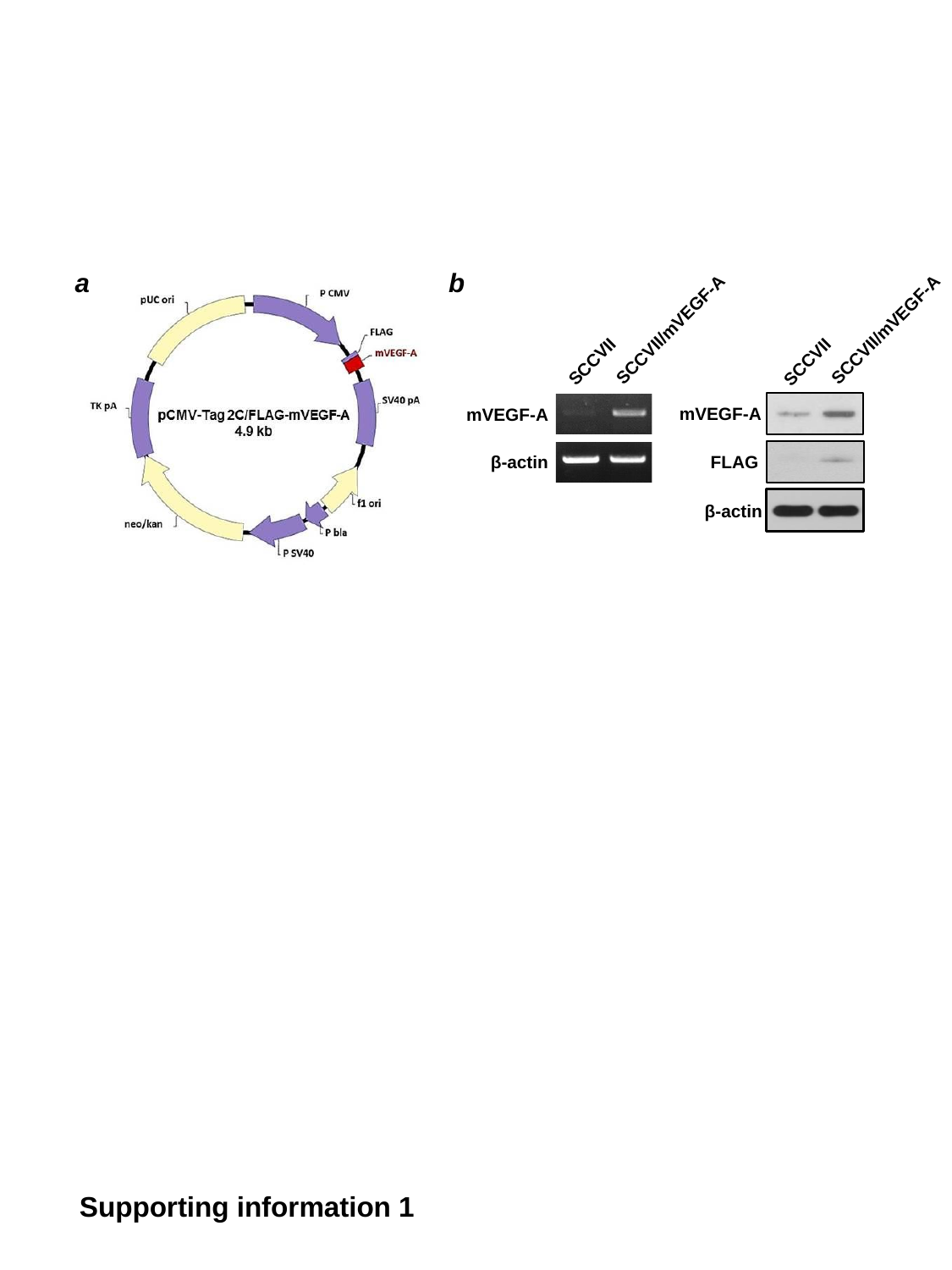

b
a
SCCVII/mVEGF-A
SCCVII/mVEGF-A
SCCVII
SCCVII
mVEGF-A
mVEGF-A
FLAG
β-actin
β-actin
Supporting information 1
